# ATG or post-transplant cyclophosphamide to prevent GVHD in matched unrelated stem cell transplantation?

**DOI:** 10.1038/s41375-024-02225-7

**Published:** 2024-03-27

**Authors:** Olaf Penack, Mouad Abouqateb, Christophe Peczynski, William Boreland, Nicolaus Kröger, Matthias Stelljes, Tobias Gedde-Dahl, Igor Wolfgang Blau, Thomas Schroeder, Urpu Salmenniemi, Alexander Kulagin, Régis Peffault de Latour, Stephan Mielke, Robert Zeiser, Ivan Moiseev, Hélène Schoemans, Christian Koenecke, Zinaida Peric

**Affiliations:** 1 Medical Clinic, Department for Haematology, Oncology and Tumorimmunology, Charité Universitätsmedizin Berlin, Berlin, Germany; 2 EBMT Transplant Complications Working Party, Paris, France; 3 EBMT Paris study office; Department of Haematology, Saint Antoine Hospital; INSERM UMR-S 938, Sorbonne University, Paris, France; 4 University Hospital Eppendorf, Hamburg, Germany; 5 University of Muenster, Muenster, Germany; 6 Oslo University Hospital, Rikshospitalet, Oslo, Norway; 7 University Hospital Essen, Essen, Germany; 8 HUCH Comprehensive Cancer Center, Helsinki, Finland; 9 RM Gorbacheva Research Institute, Pavlov University, St Petersburg, Russia; 10 Saint-Louis Hospital, BMT Unit, Paris, France; 11 Karolinska University Hospital, Stockholm, Sweden; 12 Department of Medicine I, Faculty of Medicine, Medical Centre, University of Freiburg, Freiburg, Germany; 13 Department of Hematology, University Hospitals Leuven and Department of Public Health and Primary Care, ACCENT VV, KU Leuven - University of Leuven, Leuven, Belgium; 14 Department of Hematology, Hemostasis, Oncology and Stem Cell Transplantation, Hannover Medical School, Hannover, Germany; 15 Department of Haematology, University Hospital Centre Rijeka, Rijeka, Croatia

**Keywords:** Stem-cell research, Translational research

## Abstract

There is a high risk of GVHD and non-relapse mortality (NRM) after allogeneic stem cell transplantations (alloSCT) from unrelated donors. Prophylaxis with rabbit anti-thymocyte globulin (rATG) is standard in Europe but post-transplantation Cyclophosphamide (PTCy) is an emerging alternative. We analyzed outcomes of rATG (*n* = 7725) vs. PTCy (*n* = 1039) prophylaxis in adult patients with hematologic malignancies undergoing peripheral blood alloSCT from 10/10 antigen-matched unrelated donors (MUD) between January 2018 and June 2021 in the EBMT database. The provided *P*-values and hazard ratios (HR) are derived from multivariate analysis. Two years after alloSCT, NRM in the PTCy group was 12.1% vs. 16.4% in the rATG group; *p* = 0.016; HR 0.72. Relapse was less frequent after PTCy vs. rATG (22.8% vs. 26.6%; *p* = 0.046; HR 0.87). Overall survival after PTCy was higher (73.1% vs. 65.9%; *p* = 0.001, HR 0.82). Progression free survival was better after PTCy vs. rATG (64.9% vs. 57.2%; *p* < 0.001, HR 0.83). The incidence of chronic GVHD was lower after PTCy (28.4% vs. rATG 31.4%; *p* = 0.012; HR 0.77), whereas the incidence and severity of acute GVHD were not significantly different. GVHD-free relapse-free survival was significantly higher in the PTCy arm compared to the rATG arm (2 y incidence: 51% vs. 45%; HR: 0.86 [95% CI 0.75–0.99], *p* = 0.035). In the absence of evidence from randomized controlled trials, our findings support a preference for the use of PTCy in adult recipients of peripheral blood alloSCTs from MUD.

## Introduction

One of the main clinical challenges of allogeneic stem cell transplantation (alloSCT) is its inherent non-relapse mortality (NRM) where graft-versus-host disease (GVHD) is a major contributing factor. This problem is more pronounced when using unrelated stem cell donors, leading to higher NRM than with matched-related donors [1].

In transplantations from matched unrelated stem cell donors (MUD) it has been standard of care to use rabbit anti-thymocyte globulin (rATG, also termed anti-T-cell globulin or anti-T-lymphocyte globulin; products: Grafalon® or Thymoglobulin®) in Europe to decrease the GVHD and NRM risks [2]. In the USA, the use of ATG has been less popular based on negative results of a randomized trial [3]. The prevention strategies of GVHD are currently changing. Cyclophosphamide given after alloSCT (post-transplant Cyclophosphamide, PTCy) is another option, which is now standard of care in the USA [4, 5] and is also increasingly used in some alloSCT centres in Europe.

Currently it is challenging to make sound evidence based decisions on the use of rATG or PTCy in MUD alloSCT due to the lack of large comparative data. Two randomized studies compared rATG with PTCy in the MUD setting. One randomized trial did not report any significant difference in the major outcomes of the 80 patients assigned to either PTCy or rATG prophylaxis in MUD or MRD alloSCT, however, the study was only presented at a conference, and lacked subgroup analysis for MUD alloSCT [6]. The other randomized trial was interrupted early after enrollment of 33 patients [7]. Several retrospective studies have investigated this question and one meta-analyses has pooled the results [8]. Although the evidence for prevention of GVHD varied across the studies, overall the meta-analysis indicated a lower rate of non-relapse-mortality (NRM) and a higher overall survival in MUD recipients receiving PTCy compared to those receiving rATG. Taken together the available evidence base is insufficient for clinical decision making.

To improve the evidence base, we analyzed outcomes of rATG vs. PTCy prophylaxis in adult patients with hematologic malignancies undergoing first peripheral blood alloSCT from 10/10 antigen MUD between Jan 2018 and June 2021 in the database of the EBMT.

## Patients and methods

### Study design and data collection

This is a retrospective multicenter analysis using the data set of the EBMT registry. The EBMT is a voluntary working group of more than 600 transplant centres which are required to report regular follow up on all consecutive stem cell transplantations. Audits are routinely performed to determine the accuracy of the data. The study was planned and approved by the Transplant Complications Working Party of the EBMT. All patients gave their written informed consent to use their personal information for research purposes. The study was conducted in accordance with the Declaration of Helsinki and Good Clinical Practice guidelines. Eligibility criteria for this analysis included patients older than 18 years of age at alloSCT with hematologic malignancies (acute lymphoblastic leukemia, acute myeloid leukemia, lymphoma, chronic lymphocytic leukemia, myelodysplastic syndrome or myeloproliferative neoplasms), who underwent a first alloSCT from a 10/10 antigen matched unrelated donor (MUD), from a peripheral blood stem cells source, between Jan 2018 and June 2021 in the database of the EBMT. Only patients receiving either rATG or PTCy based GVHD prophylaxis were included. Additionally, patients with more than one previous autologous transplantation, ex-vivo T-cell depletion, a combination of rATG and PTCy or use of Alemtuzumab (Campath) were not included in the study. Data collected included recipient and donor characteristics (age, sex, cytomegalovirus serostatus and Karnofsky performance status score), diagnosis and status at transplant and transplant-related factors, including conditioning regimen, stem cell source and GVHD prophylaxis. GVHD grading was performed according to published criteria for acute GVHD [9] and chronic GVHD [10]. For the purpose of this study, all necessary data were collected according to the EBMT guidelines, using the EBMT Minimum Essential Data forms.

### Statistical analysis

Median values and interquartile ranges (IQR), and minimum and maximum values were used to describe quantitative variables; frequency and percentage were used for categorical variables. Main patient-, disease-, and transplant-related characteristics were compared using Pearson’s Chi-squared test for categorical variables, and the Wilcoxon rank sum test for quantitative variables between the two groups.

Study endpoints were non-relapse mortality (NRM), overall survival (OS), progression-free survival (PFS), relapse incidence (RI), GVHD-free/relapse-free survival (GRFS), and incidence and severity of acute and chronic GVHD. The initial time was the date of transplant for all endpoints. NRM was defined as death without relapse/progression, PFS was defined as survival without relapse or progression, RI was defined as disease recurrence, GRFS was defined as survival without incidence of relapse, or grade III–IV acute GVHD, or extensive chronic GVHD. Probabilities of OS, PFS and GRFS were calculated using the Kaplan-Meier method. Cumulative incidence was used to estimate NRM, RI, as well as acute and chronic GVHD in a competing risk setting, where death and relapse were considered as competing risk as appropriate [11]. Multivariate analyses were performed using the Cox cause-specific proportional-hazards model for all endpoints. All known potential risk factors, and variables differing significantly across the groups were included in the multivariate models: patient age at transplant, year of transplant, patient and donor gender, donor to patient CMV combination, Disease Risk Index (DRI), Karnofsky Performance Status (KPS), any level of total body irradiation (TBI), conditioning intensity (RIC vs. MAC). Center effect was taken into account by introducing a random effect or ‘frailty’ into all models. Results were expressed as the hazard ratio (HR) with the 95% confidence interval (95% CI). All tests were 2-sided with a type 1 error rate fixed at 0.05. Statistical analyses were performed with R 4.3.0 software (R Development Core Team, Vienna, Austria) packages.

#### Data sharing statement

Individual participant data will not be shared because patients agreed to data sharing with EBMT as well as with publication of results, but not to share data with third parties.

## Results

### Patient characteristics

The baseline characteristics of the study population are presented in Table 1. A total of 8764 patients were included, from which 7725 (88%) received rATG, and 1039 (12%) received PTCy as GVHD prophylaxis.

**Table 1:**
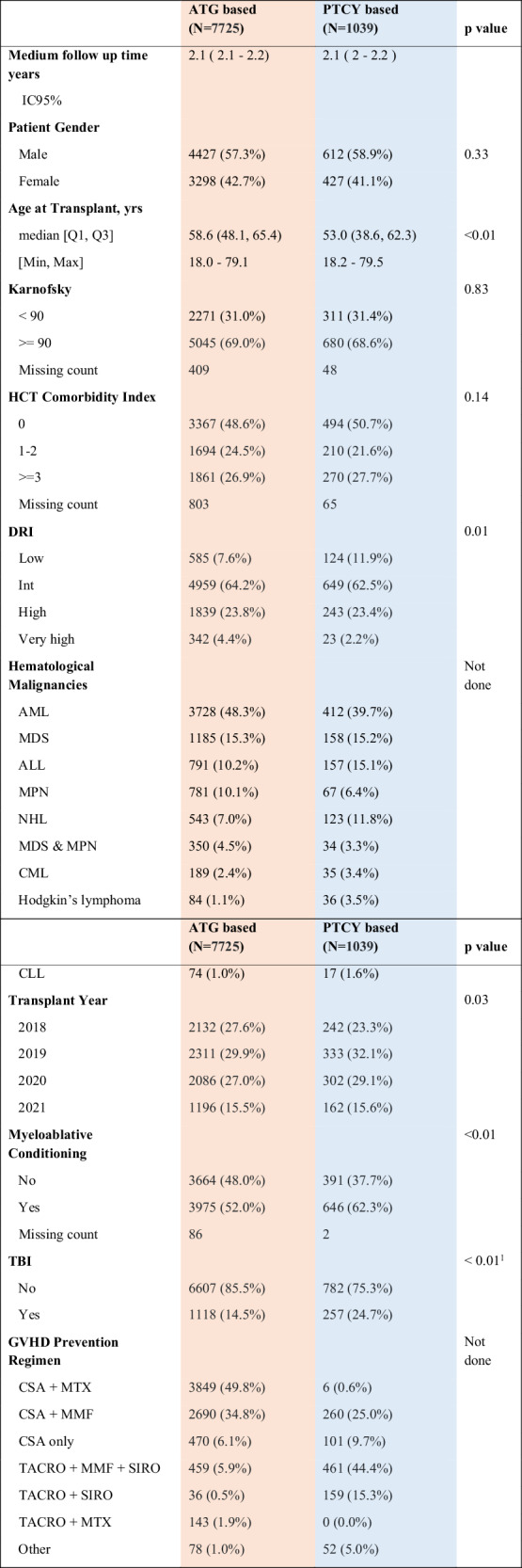
Baseline patient-, donor- and transplant-related characteristics by graft-versus-host disease prevention strategy.

Overall, the majority of patients were transplanted for acute leukemia (58%), myelodysplastic syndrome (MDS) (19.7%), myeloproliferative neoplasm (MPN) (9.7%) or lymphoma (9%). A high proportion of patients had a low/intermediate Disease Risk Index (DRI, 72.1%), and myeloablative conditioning (MAC) was more frequently performed (53.3%) than reduced intensity conditioning (RIC).

Patients in the rATG group were older, with a median age of 58.6 years (IQR (48.1, 65.4)) vs. 53 years in the PTCy group (IQR 38.6, 62.3) (*p* < 0.01), with a similar proportion of males (57.3% in rATG vs. 58.9% in PTCy, *p* = 0.33), along with a significantly lower use of TBI (14.5% vs. 24.7%, *p* < 0.01) and lower use of MAC (52% vs. 62.3%, *p* < 0.01). Also, the disease relapse index was lower and the year of transplant was more recent in the PTCy group (Table 1). The remaining parameters were balanced between the two groups. Median follow up was 2.1 years in both arms. More detailed information is given in Table 1.

### Survival, RI and NRM

**Fig. 1:**
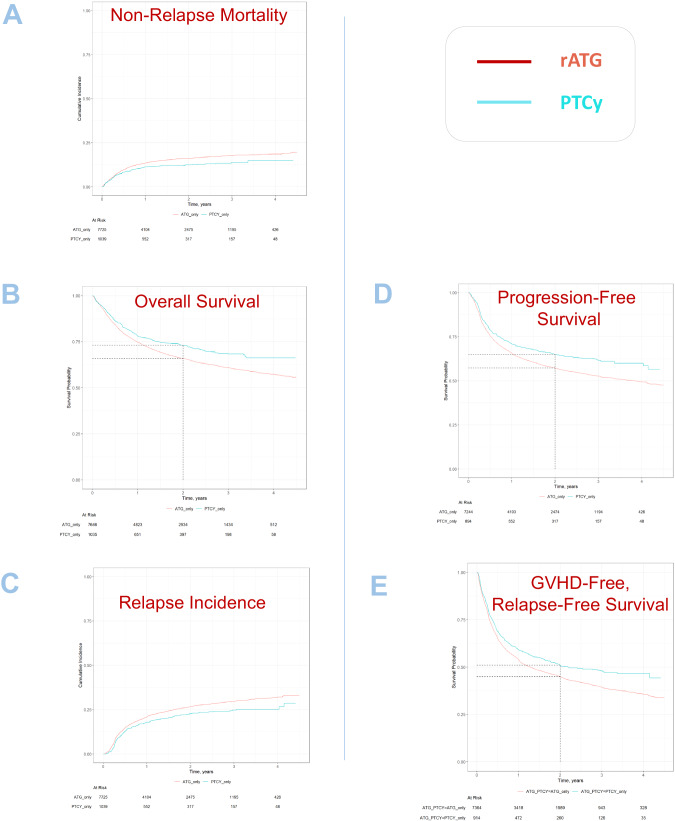
Survival outcome parameters and relapse. **A** NRM; **B** Overall survival, **C** Relapse incidence, **D** Progression-free survival and **E** GVHD-free relapse-free survival. Cumulative incidences are shown.

**Fig. 2:**
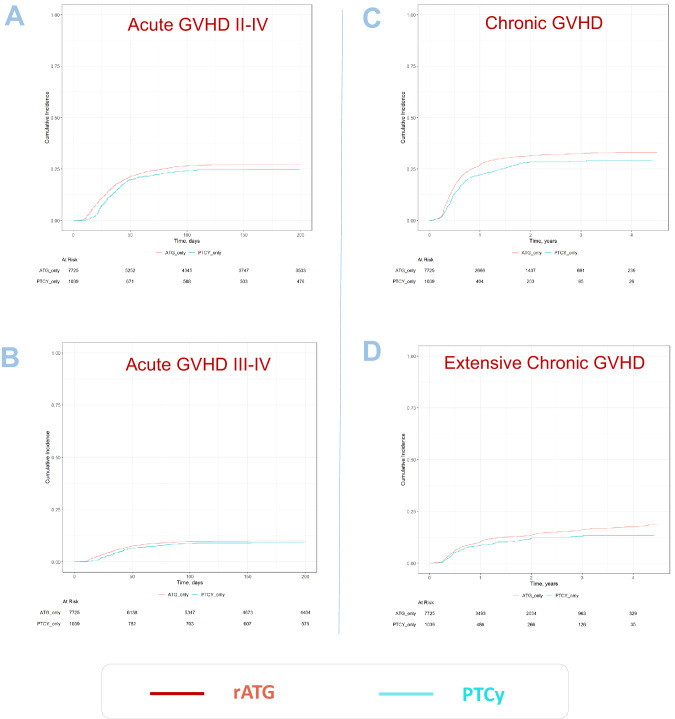
GVHD outcome parameters. **A** Acute GVHD grades II–IV; **B** Acute GVHD grades III–IV, **C** Chronic GVHD all grades and **D** Extensive chronic GVHD - Cumulative incidences are shown.

Univariate outcomes are shown in Figs. 1, 2 and Table 2. The results of the multivariate analyses are summarized in Table 3. The *P*-values and hazard ratios (HR) presented in the following results section are derived from the multivariate analysis.

**Table 2:**
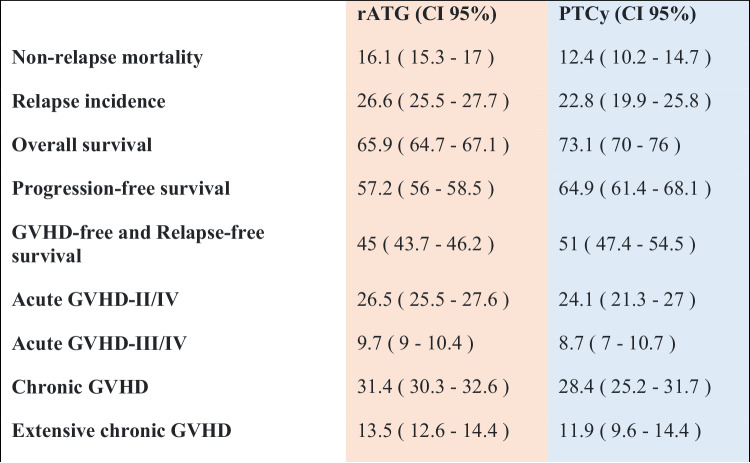
Incidence of univariate outcomes. Percentages (%) are given. All outcomes except acute GVHD are given at two years. Acute GVHD is given at day +100 after alloSCT.

**Table 3:**
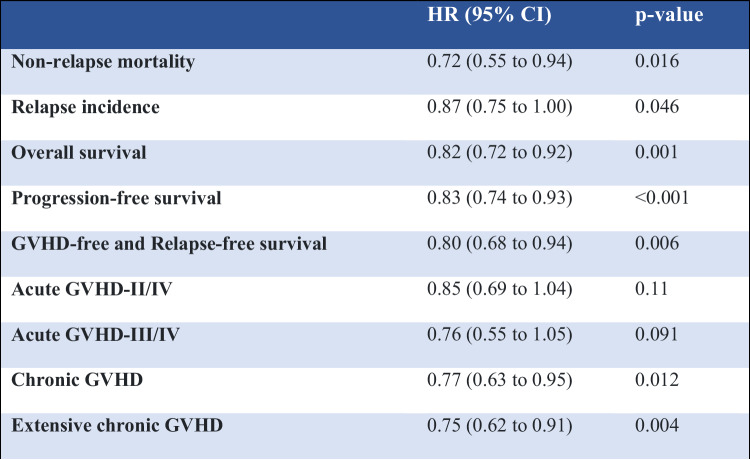
Multivariate analysis. Hazard ratios (HR) are given for PTCy with rATG being the comparator. All known potential risk factors, and variables differing significantly across the groups were included in the multivariate models: patient age at transplant, year of transplant, patient and donor gender, donor to patient CMV combination, Disease Risk Index (DRI), Karnofsky Performance Status (KPS), any level of total body irradiation (TBI), conditioning intensity (RIC vs. MAC). Center effect was taken into account by introducing a random effect or ‘frailty’ into all models.

Patients receiving PTCy had a significantly lower NRM as compared to patients receiving rATG (2 y incidence: 12.4% vs. 16.1%; HR: 0.72 [95% CI 0.55–0.94], *p* = 0.016). Similarly, OS and PFS showed a statistically significant and clinically meaningful benefit for PTCy arm, with a higher OS (2 y incidence: 73.9% vs. 65.1%; HR: 0.82 [95% CI 0.72–0.92], *p* = 0.001), and a higher PFS (2 y incidence: 64.9% vs. 57.2%; HR: 0.83 [95% CI 0.74–0.93], *p* < 0.001). RI was lower in the PTCy arm (2 y incidence: 22.8% vs. 26.6%; HR: 0.87 [95% CI 0.75–1.00], *p* = 0.046).

The causes of death are given in Table 4. No major differences between the two groups were apparent. Relapse of the underlying malignancy was the most frequent cause of death, accounting for ~50% of total deaths in both arms, followed by NRM causes: infections ~18%, GVHD ~ 16% and other alloSCT-related causes ~8% of total deaths. Secondary malignancies contributed to approximately 1% of total deaths.

**Table 4:**
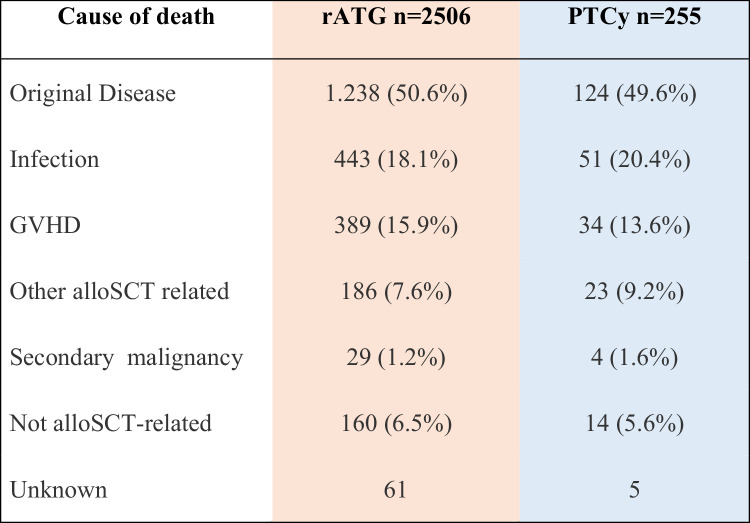
Causes of death in both cohorts. Absolute numbers and percentages are given.

### Incidence of acute and chronic GVHD, and GRFS

Overall chronic GVHD was lower in the PTCy group (2 y incidence: PTCy 28.4% vs. rATG 31.4%; HR: 0.77 [95% CI 0.63–0.95], *p* = 0.012). Extensive chronic GVHD was also reduced in patients receiving PTCy vs. rATG: (2 y incidence: 11.9% vs. 13.5%; HR: 0.75 [95% CI 0.62–0.91], *p* = 0.004).

The incidence of acute GVHD grades II-IV in patients receiving PTCy, compared to those receiving ATG was not statistically significant: (100d incidence: 24.1% vs. 26.5%; HR: 0.85 [95% CI 0.69–1.04], *p* = 0.11). Similarly, for severe acute GVHD grades III–IV (100d incidence: 8.7% vs. 9.7%; HR: 0.76 [95% CI 0.55–1.05], *p* = 0.091).

GRFS was significantly higher in the PTCy arm compared to the rATG arm (2 y incidence: 51% vs. 45%; HR: 0.86 [95% CI 0.75–0.99], *p* = 0.035).

### Incidence of neutrophil recovery and second alloSCT

The EBMT Database does not contain data on graft failure/rejection. To get insight into the initial graft’s success and any subsequent requirement for additional transplantation procedures, we investigated neutrophil recovery after the first alloSCT as well as the incidence of a second alloSCT. The median incidence of neutrophil recovery at days +30 and +60 in the ATG vs. PTCy groups was: d + 30 ATG 96% (IC95% 95.5–96.4) vs. PTCy 91% (89–92.7) and d + 60 ATG 97.9% (97.6–98.2) vs. PTCy 97.4% (96.2–98.3). The median incidence of a second alloSCT at 2 years was 4.3% (3.8–4.8) in the ATG group and 3.2% (2.2–4.6) in the PTCy group.

## Discussion

In MUD alloSCT, rATG or PTCy are often used as part of the GVHD prophylaxis strategy. In Europe, it has been standard of care to use rATG in alloSCTs with a high GVHD risk [2]. In the USA, the results of the CTN 1703 and CTN 1203 randomized trials [4, 5], demonstrating a benefit of PTCy vs. no T-cell depletion, led to a widespread use of PTCy. The present study was designed to help answering the question if PTCy or rATG should be the preferred option. In recipients of MUD alloSCT, we found that PTCy prophylaxis vs rATG prophylaxis was associated with improved NRM and overall survival.

The limitations of our current study are inherent to retrospective real world datasets, with low granularity, risk of underreporting and potential confounding factors. We observed significant differences in baseline characteristics, with the rATG group being slightly older at diagnosis and transplantation, and having received more radiation therapy. The amount of missing data was low compared to previous EBMT reports. Additionally, since the implementation of PTCY prophylaxis is a relatively recent practice, our observation period is relatively limited, this constraint our ability to draw conclusions regarding long term outcome and the occurrence of late effects. For instance, we did not observe differences in secondary malignancies but long-term follow up will be needed to answer the question if PTCy has relevant long term effects in this specific setting. We also noticed a wide variety of immunosuppressive regimens given alongside the rATG or PTCY prophylaxis, whose effect is, by design, difficult to tease out.

In the present study, we found a lower incidence of relapse among patients receiving PTCy compared to those receiving rATG. These findings raise the question of whether patients with specific tumor entities experience greater benefit from PTCy use. Future studies will need to focus on the differential impact of PTCy vs. rATG on relapse rates accross different tumor entities (e.g. lymphoid malignancies vs. myeloid neoplasms) led by disease specific working parties with access to large sets of patient data (e.g. EBMT or CIBMTR).

Further optimization of PTCy regimens for use in MUD alloSCT could potentially improve outcomes in the future. The incorporation of genetic testing and pharmacovigilance into clinical practice could be beneficial, as it has been demonstrated that polymorphisms in the genes of cyclophosphamide metabolism correlate with alloSCT outcome. Polymorphisms in major enzymes involved in cyclophospamide activation, were associated with decreased enzyme activity and a higher risk of GVHD [12]. Polymorphisms in detoxification genes lead to increased amounts of toxic metabolites and increased risk of complications [12]. Recent research suggests that refining the dosing and timing of PTCy administration as well as it’s combination with other immunosuppressive drugs could enhance its efficacy while minimizing toxicity. Based on several previous smaller studies, Ruggeri et al. evaluated 50 mg/kg PTCy on days +3 and +4 after haploidentical alloSCT along with calcineurin inhibitors and mycophenolate mofetil (MMF) from day +5 versus PTCy 50 mg/kg on days +3 and +5 and earlier start of cyclosporine A and MMF (day+1). The found a higher leukemia-free survival and a lower cGVHD incidence in the group with early start of cyclosporine A + MMF with PTCY administered on days +3 and +5 [13]. The dosing of PTCy also is a possible target to further reduce toxicity and improve efficacy as results from pre-clinical models suggest that a reduction of the standard PTCy dose (50 mg/kg per day on two days) could improve outcome. PTCy doses between 10 and 50 mg/kg/d effectively prevented fatal GVHD [14]. As a clinical translation of these results a study tested 25 mg/kg vs. 50 mg/kg PTCy given on days+3 and +4 post haploidentical alloSCT [15]. They found clinical benefits in the reduced dose group: (I) Engraftment was faster and (II) Mucositis was less severe and shorter, (III) Cytomegaly Virus (CMV) reactivation was less frequent. There were no apparent differences in major outcome parameters, such as survival or GVHD incidence but the reported follow up was relatively short and the patient population too small to detect moderate differences. Since then several groups have tested the dose reduction approach. Most recently, investigators from Sorbonne university compared outcomes with a reduced PTCy total dose (70 mg/kg) to those with the standard PT-Cy dose (100 mg/kg) in older and comorbid patients undergoing haploidentical alloSCT [16]. The reduced PTCy dose was not associated to an increased aGVHD or cGVHD risk. Engraftment was faster and the incidence of bacteremia as well as cardiac complications was lower. As a result the 2 year GVHD-free, relapse-free survival (GRFS) was higher with the reduced dose compared to the standard dose in this particular patient population. A translation of these findings to the MUD alloSCT setting as well as further optimization of combinations of PTCy with established or newer immunosuppressive drugs could bring further progress in the near future.

Taking together all the available evidence from the current study as well as from previous publications, it becomes evident that rATG and PTCy are both of clinical use in MUD alloSCT. One of the possible next steps is to investigate the combination of both strategies to further increase the efficacy in the MUD setting [17]. A combination of rATG and PTCy has been tested by several investigators in haploidentical SCT (haploSCT) [18–20]. Of note, Zhang et al. published a randomized controlled trial where on a PTCy/ATG combination or a standard-dose ATG group (‘Beijing Protocol’, ATG: 10 mg/kg) [20]. The incidence of severe aGVHD was significantly lower in the PTCy/ATG group and two-year overall survival as well as disease-free survival were improved in the PTCy/ATG group. In the setting of unrelated donor alloSCT there is less data available on the combination of PTCy and ATG. In a small trial *n* = 22 MUD alloSCT recipients were treated with a combination of PTCy and rATG and were compared to historic controls [21]. The cumulative incidence of severe aGVHD was significantly lower in the rATG/PTCy cohort but survival was not different. There is currently not enough evidence to recommend a combination of rATG with PTCy in routine clinical use in MUD alloSCT but considerable emerging data suggesting that this should be a focus area for clinical research.

In summary, we found significantly lower NRM as well as higher survival in patients with hematologic malignancies receiving peripheral blood alloSCTs from MUD when PTCy was used, as compared to rATG. The results of the current analysis build on the available evidence suggesting a preferential use of PTCy as GVHD prophylaxis in MUD alloSCT.

## Data Availability

Individual participant data will not be shared because patients agreed to data sharing with EBMT as well as with publication of results, but not to share data with third parties.
